# Investigating the associations between irritability and hot and cool executive functioning in those with ADHD

**DOI:** 10.1186/s12888-022-03818-1

**Published:** 2022-03-05

**Authors:** Silvia Colonna, Olga Eyre, Sharifah Shameem Agha, Anita Thapar, Stephanie van Goozen, Kate Langley

**Affiliations:** 1 School of Psychology, Cardiff University, Tower Building, 70 Park Place, Cardiff, CF10 3AT UK; 2 MRC Centre for Psychiatric Genetics & Genomics, Division of Psychological Medicine, School of Medicine, Cardiff University, Maindy Road, Cardiff, UK; 3 Cwm Taf Morgannwg University Health Board, Pontypridd, Wales, UK

**Keywords:** ADHD, Irritability, Executive functions

## Abstract

**Background:**

Irritability is especially pertinent to those with Attention Deficit Hyperactivity Disorder (ADHD) as it is highly prevalent and associated with a more severe clinical presentation and poorer longitudinal outcomes. Preliminary evidence suggests that top-down cognitive processes taking place in emotional contexts (i.e., hot executive functions) as opposed to those evoked in abstract scenarios (i.e., cool executive functions) may be relevant to the presentation of irritability in ADHD. This study explored the cognitive mechanisms underlying irritability in young people with ADHD, hypothesising that irritability would be associated with hot, but not cool, executive function impairments.

**Methods:**

Our sample included 219 individuals with ADHD. A composite irritability score was derived extracting items from a parent interview, with scores ranging from 0 to 5. Associations were investigated using linear regression analyses, between irritability and four hot tasks measuring sensitivity to risk, risk-taking behaviour following reward or punishment, acceptance of reward delay and reaction to unfair behaviour from others, and two cool tasks measuring set-shifting and motor inhibition.

**Results:**

As hypothesised, there were no significant associations between irritability and cool executive functions in those with ADHD; however, contrary to expectations, there was also no significant evidence that hot executive functions were associated with irritability.

**Conclusions:**

These results, in a large well characterised sample and using a comprehensive task battery, suggest that the variation in irritability in those with ADHD may not be associated with differences in hot or cool executive function performance.

**Supplementary Information:**

The online version contains supplementary material available at 10.1186/s12888-022-03818-1.

## Background

Irritability is defined as a propensity to react with anger under a minor provocation and this reaction is disproportionate compared to peers at the same developmental stage [1, 2]. Irritability is common in children and adolescents and has been linked to poor clinical [3–8] and functional outcomes [9–12] both in clinical and general population samples. Irritability is especially relevant in those with ADHD, a neurodevelopmental disorder that is characterised by symptoms of inattention, hyperactivity and impulsivity [13, 14]. Irritable symptoms are frequently observed in children with ADHD [15, 16] with a prevalence ranging between 57 and 92% [7]. Irritability in those with ADHD is associated with impairment, poor outcome and high rates of co-morbid conditions [6, 7, 17–19], and this seems to be independent of the effects of comorbidities [20–22].

ADHD is heterogeneous in terms of core symptom presentation and clinical and functional outcomes. This heterogeneity is also observed at the cognitive level; individuals with this condition do not show a specific neuropsychological profile, displaying a variety of impairments in Executive Functioning (EF) [13, 23, 24]. EF is an umbrella term that identifies higher order top-down regulatory processes involved in goal directed activities [23, 25]. EF can be split into “cool” and “hot” components. Cool EFs are used when facing abstract and decontextualized problems and include processes such as working memory and behavioural inhibition, underpinned by the dorsolateral prefrontal cortex (DLPFC) [23]. Hot EF refers to emotionally engaging cognitive processes that take place in reinforcement/motivational circumstances, such as delay aversion and risky decision making [23]. These functions are mediated by the orbital and medial prefrontal cortex (OMPFC) [23]. To date, the literature on children with ADHD has mainly focused on the cool EF; whereas recent evidence also points to the important role played by motivational and reward-related processes (hot EF) [23, 24]. Children with ADHD, compared to typically developing children, show the most robust deficits in inhibitory control, attention, working memory and vigilance for cool EF, and decision making and reward processing for hot EF [13, 14, 24]. The study of neuropsychological impairments in ADHD is particularly important to understand possible sources of heterogeneity and identify underlying risk pathways.

Neuropsychological impairments found in previous studies on those with ADHD are also common in children with high level irritability. Research on pathophysiological mechanisms of this phenotype is a new frontier and it is crucial to understand how it mediates risk for future psychiatric conditions [26], especially in those with ADHD. This ultimately could lead to the development of tailored interventions. Preliminary evidence seems to suggest the importance of impairments in reward-processing (hot EF) which might be associated with increased irritable reactions [1, 27]. In particular, children with high level irritability seem to show impairments in reward learning, reward prediction error, expected value representation and aberrant sensitivity to rewards [8, 12, 27]. However, research in this area is far from clear and findings are mixed. In particular, impairments are often found at the brain activity level but they are not supported by behavioural results [28–33]. Part of these inconsistencies might lie at the theoretical level since these previous studies used different definitions of irritability and in most cases operationalised it as categorical and severe instead of looking at the spectrum of severity [28, 29, 34, 35]. Some methodological issues are also noted in terms of type of cognitive tasks or measures used to tap reward-processing as markers of severe irritability [8, 27–29].

A few studies also suggest impairments in cool EF, such as behavioural inhibition, as markers of irritability [1, 8, 27], although results are overall not compelling [34, 36]. Notably, because of the operationalisations of irritability used in some of these previous studies, it is difficult to partial out the contribution of ADHD to these results. Thus, further research is needed to test the hypothesis that there is an association between hot EF with irritability, in those with ADHD.

Studies aiming to understand the sources of comorbidity between irritability and ADHD that focus on neuropsychological mechanisms are however scarce and far from conclusive. Previous work focused on bottom-up emotional reactivity and top-down regulatory mechanisms as possible reasons for the overlap between ADHD and severe irritability [37]. These concepts are only minimally related to hot and cool EF as both bottom-up and top-down mechanisms involve a large number of cortical and subcortical areas [37, 38] that are not completely supported by structural and functional brain abnormalities in those with ADHD [14], although a recent study suggests smaller gray matter volume in regions implicated in executive functioning associated with irritability in a community sample of children with ADHD [39]. In terms of bottom-up processes, individuals with ADHD show difficulties in both orienting attention to emotionally relevant stimuli and in the evaluation of rewards, as demonstrated by the aberrant neural activity and poor performance in delay aversions tasks [37]. This suggests an enhanced emotionality, including irritability, in ADHD [37]. Top-down mechanisms are also impaired in individuals with ADHD, as supported by studies that showed their difficulties in engaging cognitive control strategies and impairments in allocating attention away from emotional stimuli, pivotal to downregulate emotional reactivity [37]. These bottom-up and top-down impairments provide only indirect evidence for the co-occurrence of irritability in ADHD and previous work sheds doubt on whether their association is independent of ADHD symptom severity [38]. However, all these studies use different operationalisations of irritability and focused mainly on the cool EF domain, considering only delay aversion for hot EF [24, 38]. Further research is therefore needed to understand the source of co-occurrence between ADHD and irritability. Considering previous evidence on the importance of hot EF (over cool EF) for both phenotypes, tasks that comprise a broader variety of Hot EF should also be used.

Based on these knowledge gaps, it appears important to identify cognitive markers specific to irritability in those with ADHD and to understand shared mechanisms explaining their co-occurrence. To our knowledge this is the first study investigating cognitive markers of irritability in a sample of young people with ADHD. Using existing available data, the aim was to see whether irritability had a specific pattern of association with cool and hot EF. Based on previous research [1, 8, 27], we hypothesised that irritability would be independently associated with hot EF but not with cool EF in ADHD. The importance of this study lies in the possibility to understand sources of heterogeneity within ADHD and neurocognitive markers specific to irritability, ultimately investigating the impact that this phenotype has on ADHD at a cognitive level, informing future interventions.

## Methods

### Sample

The sample composed of 219 young people aged 10–18 years (mean age 14.04, s.d. 1.90). Participants were a subsample of children who took part in the Study of ADHD, Genes and Environment (SAGE) at Cardiff University (further details of this study can be seen in Stergiakouli et al., 2012) [40] and who were followed up between 2-5 years later for cognitive testing, conducted in a laboratory setting (mean 2.59; s.d. 0.91) (see [41] for further details). These individuals were originally recruited from child mental health and community paediatric clinics across the UK; all had a clinical diagnosis of ADHD. Initially, only male participants aged 14 and older with an IQ over 70 were invited to take part in the follow-up study. These criteria were then set to be inclusive of females and younger children with a broader range of IQ [41]. The mean IQ for this sample was 87.50 (s.d. 12.62), whilst 94.1% of this sample were male (*N* = 206). The majority of participants were taking medication for ADHD (78.8%) at the time of follow-up assessment, although they were asked to suspend it 24 h prior to testing.

### Clinical measures

Symptoms and research diagnoses of ADHD were assessed at baseline using the parent version of the Child and Adolescent Psychiatric Assessment (CAPA) [42]. Pervasiveness of ADHD symptoms across settings was confirmed by teachers using ChATTI reports (Child ADHD Teacher Telephone Interview) [43] or the Conner’s Teacher Rating Scale [44] at baseline.

Sample collection preceded the publication of DSM-5. Following its publication, ADHD clinical symptoms and research diagnoses at both time points were reassessed and all participants with a DSM-IV diagnosis met DSM-5 criteria for ADHD. Conduct Disorder (CD) diagnosis was formulated at follow-up using the Development and Well Being Assessment (DAWBA) [45]. All interviewers undertook comprehensive training and attended weekly supervisions with an experienced child and adolescent psychiatrist (AT).

Due to available data, and consistently with previous work [7, 46, 47], a composite score of irritability was extracted at baseline using five items from the Oppositional-Defiant Disorder (ODD) and the Depression sections of the CAPA. These items were “Losing Temper” and “Temper Tantrums” from the ODD section and “Angry or Resentful”, “Touchy or Easily Annoyed” and “Irritability” from the Depression section. The CAPA assesses irritability by investigating behaviours that are persistent and atypical compared to peers of the same developmental stage. The continuous irritability score ranged from 0 to 5 based on the presence or absence of these symptoms (*Cronbach alpha* = .63).

### Cognitive tasks

#### Cool EF

*The “Wisconsin Card Sorting Test”* (WCST) is a measure of set-shifting behaviour where participants need to identify a matching criteria to sort a set of cards [48]. These sorting criteria rely on stimulus features such as colour, form or number and keep varying after 10 consecutive trials throughout the test administration. Based on received feedback, participants need to detect changes in the matching criteria and flexibly adapt their response to keep sorting the cards properly. We used the 64-card computerised version [48]. To detect impairments in set-shifting, total number of errors and perseverative errors were considered.

*The “Go no Go” task* (GnG) is a measure of motor inhibition widely validated and extensively used in ADHD research [25], where participants are asked to inhibit a preponderant motor response. Participants need to press a button as quickly as possible in the presence of a spaceship (“go” signal) and withhold their motor response in the presence of a green planet (“no go” signal). Technical details of this task are described elsewhere [49]. We considered Reaction Time (RT) to go signals and probability of inhibition (i.e., number of commission errors to no-go stimuli, meaning participant’s response to the green planet) as dependent variables in relation to performance using participants’ dominant hand.

#### Hot EF

*The “Card Playing Task”* (CPT) is a measure of response perseveration when facing increasing loss that informs of an individual’s reward and punishment sensitivity [50]. The task involves a deck of 110 cards, divided in blocks of 10 cards. The initial block has 100% probability of winning money but the probability of losing increases by 10% with each consecutive block, such that by the end of the task, punishment completely outweighs reward. To play, participants just need to click on the deck and they receive feedback on the screen which is either “YOU WIN!” or “YOU LOSE!”. On every trial, they are asked if they are willing to keep on playing the next card or to quit the game. Participants start with no money and win or lose by £0.10, each time that a black card (spades or clubs) or a red card (hearts or diamonds) appears on the screen, respectively. When the sum of £3.10 is reached, the deck of cards starts consistently to lose and ideally that is when participants should be willing to stop. The dependent variable was the total number of cards played before quitting.

*The “Temporal Discounting Task*” (TDT) is the degree to which a reward is devaluated in relation to its temporal delay, an index of impulsivity [51, 52]. In the temporal discounting task, participants choose between a small and immediate monetary reward (ranging from £0 to £100) and a larger reward (always £100) that is however delayed either by a week, a month, a year or 2 years. Details of this task are described elsewhere [51]. The dependent variables for this task were the difference in RT between delay and immediate reward choice and the Area Under the Curve (AUC), as an index of impulsivity. AUC values range from 0 to 1; the larger AUC values, the lesser the delay discounting (i.e. less impulsivity) [53].

*The* “*Ultimatum Game*” (UG) is an economic-decision making game, often used as a measure of emotion regulation by assessing decision-making performance [54, 55]. In the UG, participants (or responders) are playing against a fictional peer opponent (the proposer) who suggests how to divide a sum of money. This proposed split varies in fairness and responders can either accept the offer and be paid accordingly or refuse it, in which case neither player gains money. Receipts of unfair offers is often associated with anger and other negative emotions leading the responder to refuse them, which is considered an irrational decision, emotionally driven [54, 55], as the responder loses the possibility of making a utilitarian choice and gaining money, albeit a small amount. Details of this task are described elsewhere [54]. The dependent variables considered were ratios of the percentages of moderately unfair (6/4; 7/3) offers accepted.

*The “Choice per Risk Task”* (CxR) is a measure of risk-taking behaviour and how it is affected by reward and punishment sensitivity [56]. The aim of the CxR is to win as many points as possible by choosing between two wheels of fortune, an experimental wheel and a control wheel, displayed randomly on the right and left side of a computer screen. The control wheel has a 50% probability of either winning or losing 1 point, whereas in the experimental wheel the chance of winning or losing either 2 or 8 points varies systematically (75% or 25%). The two wheels also differ on their relative Expected Value (∆EV), that is the difference between the control and experimental wheel that the participant is presented with, providing information on how beneficial it is to choose the experimental wheel over the control one or vice versa. Two additional positive and negative framing trial wheels are included to measure risk aversion and risk seeking. Details of this tasks are described elsewhere [56–58]. The overall propensity to gamble was chosen as dependent variable, measuring the percentage of times the experimental wheel was chosen as opposed to the control wheel.

### Sociodemographic measures

Demographic information related to child age, sex and Social Economical Status (SES) were collected at baseline. SES was assessed based on parental occupation, according to the criteria of the Standard Occupational Classification [59]. This measure has been used as a dichotomous variable, where families were categorised as having a low SES or not [60]. Age was also recorded at follow-up as well as IQ, assessed using the vocabulary and matrix reasoning tests of the Wechsler Abbreviated Scale of Intelligence (WASI) [61].

### Data analysis

Analyses were conducted using SPSS version 23 [62]. Pearson correlations were undertaken to see if executive functions were significantly associated with one another, with demographic characteristics and ADHD symptoms. Associations with categorical indicators (i.e., sex and SES) were assessed with point-biserial correlations. Multiple linear regressions were used to test the hypothesis that irritability was associated with CPT, TDT, UG, CxR (hot EF tasks) but not WCST nor GnG (cool EF tasks) performance including demographic variables as covariates. This was done in a two-stage model; in the first stage, hot and cool EF were individually regressed onto irritability; whereas in the second stage adjusted models were run controlling for demographic factors. Consistent with previous literature [63], IQ was not included as a covariate due to the variance overlap with EF measures. EF and IQ tap on very similar cognitive processes, thus its inclusion as a covariate will greatly reduce the variance associated with EF, partialling out the cognitive effects. Notably, CxR was not corrected for sex as participants with available data were all males. The Bonferroni correction was performed to account for multiple testing (*p* < .006). Parametric methods were used as the variables were all normally distributed and met all the relevant assumptions for analyses. None of the variables were skewed or kurtotic (all − 2 to + 2) according to recommendations by George and Mallery (2010) [64]. As some individuals (*n* = 18; 8.2%) did not stop their ADHD medication 24 h prior testing, sensitivity analyses were performed to see if there was a change in the results by excluding these participants (see supplementary material in Supplementary file 1). Similarly, sensitivity analyses were conducted excluding individuals with CD diagnosis, as previous work has shown its association with poor EF, over and above ADHD [54, 65–67]. Finally, sensitivity analysis was performed to explore the within time association between irritability and EF at follow-up.

## Results

Around half of this sample came from a low socioeconomic background (45.6%), as may be expected in a UK clinical ADHD sample who met ADHD diagnostic criteria at baseline to enter the study. Mean ADHD symptom scores (mean 12.5, s.d. 4.5) and ADHD diagnostic rates (80.6%) were high at follow-up. CD diagnosis was also common with a prevalence of 35%. Prevalence of irritability symptoms is shown in Table 1.

**Table 1:** Descriptive characteristics of irritability

Prevalence of irritability symptoms^a^	*N* (%)	Mean (S.D.)
“Losing Temper” item	150 (68.8%)	
“Temper Tantrum” item	193 (88.1%)	
“Angry or Resentful” item	147 (67.4%)	
“Touchy or Easily Annoyed” item	156 (71.6%)	
“Irritability” item	11 (5.0%)	
Prevalence of at least one irritability symptom	208 (95.0%)	
Mean number of Irritability symptoms		3.00 (1.23)

^a^Symptom prevalence based on their presence or absence

The presence of any irritability symptoms was common in this clinical ADHD sample, 95% of parents endorsed at least one irritability symptom (Table 1). Considering irritability as a continuum, this clinical ADHD sample endorsed 3 symptoms on average (Table 1).

### Association between EF tasks and irritability

The cool EFs were significantly associated with demographic characteristics as shown in Table 2. Counter to this, EF measures did not seem to be associated with one another with the following exceptions: WCST total errors were significantly associated with WCST perseverative errors (*r* = .78, *p* < .001) and with CxR propensity to gamble (*r* = .20, *p* = .02); TDT RT was significantly associated with TDT AUC (*r* = −.22, *p* = .003). UG Moderately Unfair offers accepted significantly correlated with CPT total cards played (*r* = .32, *p* = .001). ADHD symptoms at follow-up also did not show any significant associations with EF measures whereas it was significantly associated with irritability (*r* = 0.18, *p* = 0.01).

**Table 2:** Correlations between EF measures with Age,SES and ADHD symptoms

EF measures	Age	SES (Low)	ADHD symptoms
Cool EFs			
WCST Total errors	−0.18^*^	0.19^*^	0.07
WCST Perseverative errors	−0.19^*^	0.19^*^	0.01
GnG RT to go signals	−0.28**	0.05	0.12
GnG Probability of inhibition	0.36^**^	−0.21^**^	−0.08
Hot EFs			
CPT Total number of Cards	−0.09	0.07	0.04
TDT RT (delayed -immediate choice)	0.01	0.11	0.004
TDT AUC	0.06	−0.07	−0.01
UG Moderately Unfair offers accepted	0.03	−0.21^*^	−0.19
CxR propensity to gamble	−0.08	−0.03	0.02

*SES* Socioeconomic status, *WCST* Wisconsin Card Sorting Test task, *GnG* Go/no-Go task, *CPT* Continuous Performance Task, *TDT* Temporal Discounting Task, *UG* Ultimatum Game, *RT* Reaction Time, *AUC* Area Under the Curve

**significant at *p* < 0.01

*significant at *p* < 0.05

As shown in Table 3, overall childhood irritability was not associated with hot (CPT, TDT, UG, CxR) or cool (WCST and GnG) EF in adolescents with ADHD. This was consistent when controlling for age, sex and SES. A significant association between irritability in childhood and WCST perseverative error (cool EF measure) in adolescence did not withstand Bonferroni correction.

**Table 3:** Pattern of associations between irritability and Hot and Cool EF measures

	*N*	Model	Standardised Beta	Unstandardized Beta (95% CI)	*p*-value
Cool EFs					
WCST Total Errors	*N* = 173	Unadjusted	B = 0.11	B = 0.64 (− 0.29; 1.57)	*p* = 0.17
		Adjusted	B = 0.09	B = 0.51 (−0.40; 1.41)	*p* = 0.27
WCST Perseverative Errors	*N* = 173	Unadjusted	B = 0.22	B = 0.70 (0.20; 1.20)*	*p* = 0.01
		Adjusted	B = 0.19	B = 0.62 (0.13; 1.11)*	*p* = 0.01
GnG RT to go signals	*N* = 185	Unadjusted	B = 0.05	B = 1.91 (−3.86; 7.67)	*p* = 0.52
		Adjusted	B = 0.03	B = 1.04 (−4.54; 6.62)	*p* = 0.71
GnG Probability of inhibition	*N* = 185	Unadjusted	B = −0.04	B = − 0.68 (−3.23; 1.87)	*p* = 0.60
		Adjusted	B = 0.03	B = 0.55 (−1.74; 2.84)	*p* = 0.64
Hot EFs					
CPT total number of Cards	*N* = 208	Unadjusted	B = 0.02	B = 0.49 (−3.36; 4.33)	*p* = 0.80
		Adjusted	B = 0.01	B = 0.14 (−3.77; 4.04)	*p* = 0.95
TDT RT (delayed -immediate choice)	*N* = 176	Unadjusted	B = −0.10	B = −12.6 (−32.0; 6.86)	*p* = 0.20
		Adjusted	B = −0.12	B = −14.4 (−34.0.;5.29)	*p* = 0.15
TDT AUC	*N* = 176	Unadjusted	B = − 0.13	B = − 0.02 (− 0.05; 0.004)	*p* = 0.10
		Adjusted	B = − 0.12	B = − 0.02 (− 0.05; 0.01)	*p* = 0.14
UG Moderately Unfair offers accepted	*N* = 116	Unadjusted	B = − 0.19	B = − 0.05 (− 0.1; − 0.001)	*p* = 0.05
		Adjusted	B = − 0.18	B = − 0.05 (− 0.10; 0.004)	*p* = 0.70
CxR propensity to gamble	*N* = 151	Unadjusted	B = 0.09	B = 0.006 (−.01; .02)	*p* = 0.30
		Adjusted	B = 0.09	B = 0.005 (−.01; .02)	*p* = 0.34

Adjusted models were corrected for Age, sex, SES, where possible

*WCST* Wisconsin Card Sorting Test task, *GnG* Go/no-Go task, *CPT* Continuous Performance Task, *TDT* Temporal Discounting Task, *UG* Ultimatum Game, *RT* Reaction Time, *AUC* Area Under the Curve

*Significant *p*-values

Sensitivity analyses excluding adolescents who had not withdrawn their medication, those with CD diagnosis at follow-up, and exploring within time associations between irritability and EF in adolescence did not alter the findings (see supplementary materials in Supplementary file 1).

## Discussion

This is the first study testing the hypothesis of an association between irritability and hot executive functioning in those with ADHD. Overall, results suggest that irritability is not associated with hot EFs as opposed to cool EFs. This was independent of the role played by demographic factors, suggesting that irritability per se does not seem to be associated with hot or cool EF performance in a sample of young people with ADHD.

This study adds to previous research in the field of ADHD and neurocognitive markers of irritability. The results support those studies that failed to find significant behavioural differences comparing children with high level irritability and controls on cognitive performance [28–31, 33], countering the hypothesis that irritability is associated with hot EFs. They are also consistent with previous research in ADHD populations where irritability failed to show an association with cognitive markers, over and above ADHD symptom severity [38]. These results are however in contrast with previous evidence in favour of this theory linking irritability and hot cognitive functions [8, 12, 27]. It should be noted that previous research focused on a narrow range of hot cognitive tasks or used tasks that did not actually tap hot cognitive processing [28, 29]. Conversely this current study used a broad range of well validated measures to assess cool and hot EF. It is therefore possible that the different results obtained reflect a more comprehensive assessment that enables a greater insight on the impact of irritability on cognitive performance. Previous significant results were in children with severe irritability defined in a manner that strongly overlapped with ADHD [28, 29, 34, 35]. Thus, it is possible that previously observed associations between severe irritability and hot executive functioning might have actually been driven by ADHD. This is further confirmed by previous findings suggesting that ADHD and severe irritability are associated with the same cognitive parameters [38]. Finally, previous research on cognitive markers of irritability generally focused on a more severe and categorical operationalisation of this phenotype as opposed to using a broader and continuous measure.

These non-significant findings also inform the debate on irritability [68] by highlighting that, in a sample of young people with ADHD, this phenotype may actually be a core feature of ADHD rather than indexing heterogeneity from a cognitive perspective. Nearly all individuals in this sample had at least one irritability symptom. Moreover, genetic studies suggest that ADHD genetic liability as indexed by polygenic risk scores also predict irritability [69]. If irritability is a core feature of ADHD, then it will be challenging if not impossible to detect association with cognitive processes because of limited variation within an ADHD only sample. However, there could be other pathophysiological mechanisms associated with irritability that might be investigated in the future in an attempt to assess the relevance of this phenotype at the cognitive level in those with ADHD.

One possible additional area of investigation, not assessed as part of this study, is Frustrative Non-Reward (FNR). As suggested by previous reviews [1, 8, 27], irritability is intertwined with the concept of FNR and considers those showing high level irritability as having a lower tolerance and an aberrant response to frustration. Thus, it is possible that cognitive markers of irritability may be particularly connected to this concept of FNR, as opposed to broader deficits in reward processing, as explored in this study. Previous studies investigating individuals responses to blocked reward attainment, with tasks specifically designed to induce frustration, showed consistent findings; compared to controls, children with high level irritability display a greater emotional response to frustration with a negative impact on cognitive performance, supported both at neurophysiological and behavioural level [35, 70]. These neurophysiological results are also supported in a study of kindergarten aged children with externalising problems [71]. Additionally, the EF model is only one of the disrupted neuropsychological paradigms of ADHD [25, 72–75]. Thus, future research on the overlap between ADHD and irritability could investigate other pathophysiological mechanisms to test the impact of this phenotype in youths with ADHD.

This study has several strengths. It is innovative as it is the first study looking at cognitive markers of irritability in ADHD, exploring potential pathological mechanisms leading to impairment in this population. It benefits from a clear and circumscribed operationalisation of irritability, as opposed to looking at facets of this construct (e.g., trait anger, emotional lability, or dysregulation). A wide range of well validated cognitive tasks was also used, ultimately enhancing the opportunity to directly compare these two cognitive aspects. Nonetheless, the results of this study should be considered in light of several limitations. Firstly, this is a post hoc study and the measures available were originally chosen to address a different aim. Thus, a comprehensive scale specifically designed to tap irritability could not be used. Additionally, the EF tasks were initially selected as well validated in identifying variance in those with ADHD population and not as direct measures of cognitive markers of irritability. Secondly, this study lacks both a comparison and a control group which would complement the study of the cognitive markers of irritability by looking at its impact on hot cognitive functioning both in the general population and in other clinical samples. The lack of typically developing controls also limits the possibility to compare EF performances, ultimately being unable to refine these analyses to those actually showing EF impairments. Thirdly, despite the large number of families with ADHD in this study at baseline, participant drop-out, protocol changes and quality control checks negatively impacted the availability of cognitive data at follow-up. Thus, investigating the same research questions in a larger sample might enable more definitive conclusion. Fourthly, irritability tends to decrease with age [8], thus exploring the associations between irritability and hot cognitive functioning within time, might increase the power to detect an effect. Due to the data availability, longitudinal associations were investigated in this study. It is also possible that the wide age range of this current sample can have biased these results at least in respect to certain tasks. Although irritability was not associated with age, age was correlated with cognitive performance on GnG and WCST. Similarly, our sample was largely composed of males therefore it could be that the inclusion of females might lead to different results although, irritable symptoms prevalence does not seem to vary based on sex [4, 47, 76, 77]. Finally, whilst this study did consider the role of CD, data on other co-occurring conditions (e.g. depression, anxiety) was not available and so could not be explored.

## Conclusions

In conclusion this is the first study investigating an association between reward-related impairments in the form of hot EF and a dimensional measure of irritability in a clinical sample of young people with ADHD, as suggested by previous work and theoretical rationale. Despite the broad range of well validated hot and cool EF measures used, our results failed to support our initial hypothesis; irritability does not seem to be associated with impairments in reward processing, in youths with ADHD. Other neuropsychological processes (e.g., FNR) may be more relevant to the associations between ADHD and irritability than hot and cool EF and should be investigated. Irritability is a cross-diagnostic symptom, thus investigating its cognitive markers in both externalising and internalising conditions could give better insight about pathophysiological mechanisms of this phenotype across different cohorts. Addressing these aspects in future research could improve the theoretical models of ADHD and provide a better understanding of the importance of irritability across conditions, being ultimately useful in clinical practice.

## Supplementary Information

**Additional file 1: Table S1.** Pattern of associations between irritability and hot and cool EF measures. **Table S2.** Pattern of associations between irritability and hot and cool EF measures. **Table S3.** Correlations between irritability measured in adolescence and executive functions. **Table S4.** Correlation matrix between EF measures, IQ and Irritability.

## Data Availability

The datasets generated and/or analysed during the current study are not publicly available due the sensitive nature of data and ethical approval constraints but are available from the corresponding author on reasonable request.

## Abbreviations

ADHD: Attention Deficit Hyperactivity Disorder
CAPA: Child and Adolescent Psychiatric Assessment
CD: Conduct Disorder
CPT: Card Playing Task
CxR: Choice per Risk Task
DAWBA: Development and Well-being Assessment
DSM: Diagnostic and Statistical Manual of Mental Disorders
EFs: Executive Functions
GnG: Go no Go
IQ: Intelligence Quotient
ODD: Oppositional Defiant Disorder
RT: Reaction Time
SES: Social Economic Status
TDT: Temporal Discounting Task
UG: Ultimatum Game
WCST: Wisconsin Card Sorting Test
