# Supplementary material for: Investigating the associations between irritability and hot and cool executive functioning in those with ADHD
Source: BMC Psychiatry. 2022 Mar 5;22:166. doi: 10.1186/s12888-022-03818-1 (PMC8898423; doi:10.1186/s12888-022-03818-1)
Supplement: Supplementary file 1 — Additional file 1: Table S1. Pattern of associations between irritability and hot and cool EF measures. Table S2. Pattern of associations between irritability and hot and cool EF measures. Table S3. Correlations between irritability measured in adolescence and executive functions. Table S4. Correlation matrix between EF measures, IQ and Irritability. [file 12888_2022_3818_MOESM1_ESM.docx]

**SUPPLEMENTARY MATERIALS**

The following table shows the results of the same set of analyses, previously performed on our sample of young people with ADHD (N = 219), excluding 18 participants who did not suspend their medication 24h prior testing (n= 201).

S Table 1 Pattern of associations between irritability and hot and cool EF measures.

|  | N | Model | Standardized Beta | Unstandardized Beta (95% CI) | p-value |
| --- | --- | --- | --- | --- | --- |
| Cool EFs | | | | | |
| WCST Total errors | N= 162 | Unadjusted | B=0.12 | B= 0.68 (-0.26; 1.62) | p= 0.18 |
|  |  | Adjusted | B=0.11 | B= 0.62 (-0.30; 1.55) | p= 0.19 |
| WCST Perseverative errors | N= 162 | Unadjusted | B=0.24 | B= 0.73 (0.24; 1.23)** | p= 0.004 |
|  |  | Adjusted | B=0.22 | B= 0.69 (0.20; 1.17)* | p= 0.01 |
| GnG RT to go signals | N= 174 | Unadjusted | B=0.05 | B= 1.82 (-3.98; 7.63) | p= 0.54 |
|  |  | Adjusted | B=0.03 | B= 1.08 (-4.54; 6.70) | p= 0.71 |
| GnG Probability of inhibition | N= 174 | Unadjusted | B=-0.05 | B= -0.76 (-3.36; 1.84) | p= 0.52 |
|  |  | Adjusted | B=0.04 | B= 0.57 (-1.77; 2.91) | p= 0.63 |
| Hot EFs | | | | | |
| CPT total number of Cards | N= 191 | Unadjusted | B=0.02 | B= 0.51 (-3.39; 4.42) | p= 0.80 |
|  |  | Adjusted | B=0.003 | B= 0.09 (-3.86; 4.03) | p= 0.97 |
| TDT RT (delayed - immediate choice) | N= 164 | Unadjusted | B=-0.10 | B= -11.7 (-31.5; 8.11) | p= 0.25 |
|  |  | Adjusted | B=-0.11 | B= -13.6 (-33.7; 6.55) | p= 0.18 |
| TDT AUC | N= 164 | Unadjusted | B=-0.15 | B= -0.02 (-0.05; 0.001) | p= 0.06 |
|  |  | Adjusted | B=-0.15 | B= -0.02 (-0.05; 0.002) | p= 0.07 |
| UG Moderately Unfair offers accepted | N= 107 | Unadjusted | B=-0.18 | B= -0.05 (-0.10; 0.01) | p= 0.08 |
|  |  | Adjusted | B=-0.17 | B= -0.05 (-0.10; 0.01) | p= 0.10 |
| CxR propensity to gamble | N= 142 | Unadjusted | B=0.09 | B= 0.01 (-0.01; 0.02) | p= 0.35 |
|  |  | Adjusted | B=0.08 | B= 0.01 (-0.01; 0.02) | p= 0.40 |

* Significant results

WCST = Wisconsin Card Sorting Test task, GnG = Go/no-Go task, CPT = Continuous Performance Task, TDT = Temporal Discounting Task, UG = Ultimatum Game. RT = Reaction Time, AUC = Area Under the Curve.
Adjusted models were corrected for age, sex, SES, where possible.

The following table shows the results of the same set of analyses, previously performed on our sample of young people with ADHD (N = 219), excluding 72 participants who had CD diagnosis at follow-up (n= 147).

S Table 2 Pattern of associations between irritability and hot and cool EF measures.

|  | N | Model | Standardised beta | Unstandardized Beta (95% CI) | p-value |
| --- | --- | --- | --- | --- | --- |
| Cool EFs | | | | | |
| WCST Total errors | N= 113 | Unadjusted | B= 0.07 | B= 0.36 (-0.72; 1.43) | p= 0.52 |
|  |  | Adjusted | B= 0.05 | B= 0.30 (-0.75; 1.34) | p= 0.58 |
| WCST Perseverative errors | N= 113 | Unadjusted | B= 0.21 | B= 0.57 (0.05; 1.09)* | p= 0.03 |
|  |  | Adjusted | B= 0.19 | B= 0.52 (0.01; 1.03) | p= 0.05 |
| GnG RT to go signals | N= 131 | Unadjusted | B= 0.05 | B= 1.63 (-4.64; 7.89) | p= 0.61 |
|  |  | Adjusted | B= 0.04 | B= 1.25 (-4.68; 7.17) | p= 0.68 |
| GnG Probability of inhibition | N= 131 | Unadjusted | B= -0.04 | B= -0.61 (-3.53; 2.32) | p= 0.68 |
|  |  | Adjusted | B= 0.03 | B= 0.41 (-2.11; 2.93) | p= 0.75 |
| Hot EFs | | | | | |
| CPT total number of Cards | N= 139 | Unadjusted | B= -0.05 | B= -1.15 (-5.46; 3.17) | p= 0.60 |
|  |  | Adjusted | B= -0.05 | B= -1.29 (-5.65; 3.06) | p= 0.56 |
| TDT RT (delayed - immediate choice) | N= 122 | Unadjusted | B= -0.05 | B= -6.05 (-27.3; 15.2) | p= 0.58 |
|  |  | Adjusted | B= -0.06 | B= -7.09 (-28.8; 14.6) | p= 0.52 |
| TDT AUC | N= 122 | Unadjusted | B= -0.14 | B= -0.02 (-0.05; 0.01) | p= 0.15 |
|  |  | Adjusted | B= -0.12 | B= -0.02 (-0.05; 0.01) | p= 0.21 |
| UG Moderately Unfair offers accepted | N= 77 | Unadjusted | B= -0.20 | B= -0.05 (-0.12; 0.01) | p= 0.10 |
|  |  | Adjusted | B= -0.21 | B= -0.06 (-0.12; 0.01) | p= 0.08 |
| CxR propensity to gamble | N= 105 | Unadjusted | B= 0.01 | B= 0.00 (-0.01; 0.01) | p= 0.94 |
|  |  | Adjusted | B= -0.01 | B= 0.00 (-0.01; 0.01) | p= 0.96 |

* Significant results

WCST = Wisconsin Card Sorting Test task. GnG = Go/no-Go task. CPT = Continuous Performance Task. TDT = Temporal Discounting Task. UG = Ultimatum Game. RT = Reaction Time. AUC = Area Under the Curve.
Adjusted models were corrected for age. sex. SES. where possible.

The following table shows the results of within time correlations between irritability and executive functions both measured in adolescence.

Notably, as mentioned in the method section of this paper, clinical symptoms and diagnosis were assessed at follow-up using the DAWBA as opposed to the CAPA. This reduced the availability of data on irritability in adolescence and further impacted on the number of individuals with available cognitive data (see S Table 3). The total sample size at follow-up consisted of 149 adolescents with an ADHD diagnosis in childhood.

Irritability composite score at follow-up consisted of summing up responses with a score of 2 (i.e., “A lot more than others”) on the following items of the DABA: “temper outbursts”, “angry or resentful” and “easily annoyed”. Irritability scores ranged from 0 to 3, with a mean of 1.53 (s.d. 1.28).

S Table 3 Correlations between irritability measured in adolescence and executive functions.

|  | Irritability in adolescence | | |
| --- | --- | --- | --- |
| EF measures | N | Pearson coefficient | p-value |
| Cool EF | | | |
| WCST Total errors | N= 129 | -0.02 | 0.82 |
| WCST Perseverative errors | N= 129 | 0.02 | 0.83 |
| GnG RT to go signals | N= 132 | -0.08 | 0.35 |
| GnG Probability of inhibition | N= 132 | -0.01 | 0.93 |
| Hot EF | | | |
| CPT total number of Cards | N= 149 | 0.11 | 0.17 |
| TDT RT (delayed - immediate choice) | N= 124 | -0.07 | 0.45 |
| TDT AUC | N= 124 | -0.06 | 0.49 |
| UG Moderately Unfair offers accepted | N= 54 | 0.01 | 0.96 |
| CxR propensity to gamble | N= 138 | -0.02 | 0.79 |

WCST = Wisconsin Card Sorting Test task. GnG = Go/no-Go task. CPT = Continuous Performance Task. TDT = Temporal Discounting Task. UG = Ultimatum Game. RT = Reaction Time. AUC = Area Under the Curve. EF = Executive functions.

S Table 4 Correlation matrix between EF measures, IQ and Irritability

|  | Irritability | WCST Total errors | WCST Perseverative errors | GnG RT to go signals | GnG Probability of inhibition | CPT total number of Cards | TDT RT (delayed - immediate choice) | TDT AUC | UG Moderately Unfair offers accepted | CxR propensity to gamble | IQ |
| --- | --- | --- | --- | --- | --- | --- | --- | --- | --- | --- | --- |
| Irritability | 1 |  |  |  |  |  |  |  |  |  |  |
| WCST Total errors | 0.11 | 1 |  |  |  |  |  |  |  |  |  |
| WCST Perseverative errors | 0.22** | 0.78** | 1 |  |  |  |  |  |  |  |  |
| GnG RT to go signals | 0.05 | 0.10 | 0.06 | 1 |  |  |  |  |  |  |  |
| GnG Probability of inhibition | -0.04 | -0.03 | -0.05 | -0.14 | 1 |  |  |  |  |  |  |
| CPT total number of Cards | 0.02 | 0.03 | 0.04 | 0.15 | -0.06 | 1 |  |  |  |  |  |
| TDT RT (delayed - immediate choice) | -0.10 | -0.03 | -0.06 | 0.03 | -0.02 | 0.07 | 1 |  |  |  |  |
| TDT AUC | -0.13 | -0.12 | -0.13 | -0.004 | -0.04 | 0.02 | -0.22** | 1 |  |  |  |
| UG Moderately Unfair offers accepted | -0.19* | -0.02 | -0.03 | 0.12 | 0.11 | 0.32** | -0.04 | -0.09 | 1 |  |  |
| CxR propensity to gamble | .091 | .203* | .126 | .033 | -.068 | .083 | .003 | .020 | -.092 | 1 |  |
| IQ | -0.24** | -0.35** | -0.33** | -0.10 | 0.06 | -0.02 | -0.04 | -0.02 | 0.11 | -0.18* | 1 |

*significant results p<0.05, ** significant results p =< 0.01

WCST = Wisconsin Card Sorting Test task. GnG = Go/no-Go task. CPT = Continuous Performance Task. TDT = Temporal Discounting Task. UG = Ultimatum Game. RT = Reaction Time. AUC = Area Under the Curve. EF = Executive functions.
